# Socioeconomic and Geographic Disparities in Health Information Seeking and Internet Use in Puerto Rico

**DOI:** 10.2196/jmir.2007

**Published:** 2012-07-19

**Authors:** Lila J Finney Rutten, Bradford W Hesse, Richard P Moser, Ana Patricia Ortiz Martinez, Julie Kornfeld, Robin C Vanderpool, Margaret Byrne, Guillermo Tortolero Luna

**Affiliations:** ^1^Clinical Monitoring Research ProgramSAIC-Frederick, IncNational Cancer Institute at FrederickFrederick, MDUnited States; ^2^Behavioral Research ProgramHealth Communications and Informatics Research BranchNational Cancer InstituteBethesda, MDUnited States; ^3^Behavioral Research ProgramScience of Research and Technology BranchNational Cancer InstituteBethesda, MDUnited States; ^4^Comprehensive Cancer CenterMedical Sciences CampusUniversity of Puerto RicoSan JuanPuerto Rico; ^5^University of MiamiMiller School of Medicine, Department of Epidemiology and Public HealthMiami, FLUnited States; ^6^College of Public HealthUniversity of KentuckyLexington, KYUnited States; ^7^University of MiamiMiami, FLUnited States

**Keywords:** Health information seeking, cancer information seeking, Internet use, disparities, special populations, geographic trends

## Abstract

**Background:**

Geographically isolated Hispanic populations, such as those living in Puerto Rico, may face unique barriers to health information access. However, little is known about health information access and health information-seeking behaviors of this population.

**Objective:**

To examine differences in health and cancer information seeking among survey respondents who ever used the Internet and those who did not, and to explore sociodemographic and geographic trends.

**Methods:**

Data for our analyses were from a special implementation of the Health Information National Trends Survey conducted in Puerto Rico in 2009. We collected data through random digit dialing, computer-assisted telephone interviews (N = 639). The sample was drawn from the eight geographic regions of the Puerto Rico Department of Health. To account for complex survey design and perform weighted analyses to obtain population estimates, we analyzed the data using SUDAAN. Frequencies, cross-tabulation with chi-square, and logistic regression analyses were conducted. Geographic information system maps were developed to examine geographic distributions of Internet use and information seeking.

**Results:**

Of 639 participants, 142 (weighted percentage 32.7%) indicated that they had ever gone online to access the Internet or World Wide Web; this proportion was substantially lower than that of US mainland Hispanics who reported using the Internet (49%). While 101 of 142 (weighted percentage 59.6%) respondents who used the Web had ever sought health information, only 118 of 497 (weighted percentage 20.0%) of those who did not use the Web had sought health information. The pattern was similar for cancer information: 76 of 142 respondents (weighted percentage 47.2%) who used the Web had ever sought cancer information compared with 105 of 497 (weighted percentage 18.8%) of those who had not used the Web. These results were slightly lower but generally consistent with US mainland Hispanics’ health (50.9%) and cancer (26.4%) information seeking. Results of separate logistic regression models controlling for sociodemographic characteristics demonstrated that, compared with individuals who did not seek health or cancer information, those who did were over 5 times as likely to have used the Internet (odds ratio 5.11, *P *< .001). Those who sought cancer information were over twice as likely to have used the Internet (odds ratio 2.5, *P *< .05). The frequency of Internet use and health and cancer information seeking was higher in the San Juan metro region than in more rural areas.

**Conclusions:**

Our results contribute to the evidence base for health and cancer communication planning for Puerto Rico, and suggest that health education and outreach efforts should explore the use of available and trusted methods of dissemination such as radio and television, as well as community-based health care providers and organizations, to supplement and encourage use of the Internet as a source of health information.

## Introduction

The Internet has become a valuable tool in supporting consumers’ health information and health care needs [1-4] and is used for health information seeking by millions of people worldwide [3]. As access to the Internet has increased, and as the availability of health information has expanded, online health information seeking has increased [3]. National Healthy People 2020 objectives for health communication and health information technology in the United States are directed toward the overarching goal to “use health communication strategies and health information technology to improve population health outcomes and health care quality, and to achieve health equity” [5]. The Healthy People 2020 objectives for health communication and health information technology draw on an expanding body of empirical evidence that documents an association between greater access to health information through the Internet and improved health knowledge, attitudes, and behavior [5-9].

Despite the building of momentum and support for improving access to health information, it remains to be seen whether the benefits of online access will be equitably enjoyed by consumers, particularly among traditionally underserved and disadvantaged populations in the United States [3]. Previous studies with national survey data have documented profound subpopulation disparities in access to health information, wherein persons with lower socioeconomic status or lower levels of education appear to face greater barriers to obtaining health information than those with higher socioeconomic status and education [10-14].

Disparities in information access in the United States may be particularly significant for Hispanic populations, who often face additional barriers in terms of language, culture, and media use [10-12,15,16]. Despite the proliferation of the Internet, mobile technology, and wireless devices, research demonstrates the persistence of a digital divide between Hispanic populations in the mainland United States and other groups, wherein less than one-third of Spanish-speaking Hispanics use the Internet, compared with 71% in the non-Hispanic population [13]. Geographically isolated Hispanic populations, such as those living in Puerto Rico, may face additional or unique barriers to health information access [17]. However, little is known about health information access and health information-seeking behaviors of this population.

Health information seeking has been shown to increase individuals’ involvement in health-related decision making and improve their satisfaction with their health choices [10]. Information has been demonstrated to improve individuals’ coping abilities, reduce anxiety, alleviate mood disturbances, and improve communication with family members [10]. In Puerto Rico, cancer is the second-leading cause of death, and significant disparities in cancer incidence, mortality, and survival for several cancer types have been documented in this US territory [18-24], some of which may be partly influenced by health information access. For example, breast cancer mortality rates for certain cancers vary by region and by socioeconomic status in Puerto Rico. This disparity may be due to both lack of information and lack of access to information about state-of-the-art treatment options.

Puerto Rico is an island of approximately 3.7 million people with approximately one-third of the population concentrated in the San Juan metropolitan area and its adjacent municipalities [25]. Over 44% of Puerto Ricans live in poverty; this is nearly double the proportion of Puerto Rican-origin Hispanics living in poverty in the United States (24%) [25].

Prior research has documented socioeconomic disparities across the municipalities, including disparities in socioeconomic indicators such as the proportion of residents living below the poverty level and the proportion of residents with lower levels of education [24]. Poverty rates range from approximately 35% in affluent areas near San Juan to 77.8% in rural areas in the geographical center of the island [26]. Specifically, greater socioeconomic deprivation has been observed in the centrally located municipalities of the island relative to the municipalities in the capital city, San Juan, metropolitan area [24].Such socioeconomic disparities have been shown to coincide with cancer morbidity and mortality [24,27], as well as access to and use of health information [10-14]. Previous research in Puerto Rico has documented socioeconomic disparities in incidence and mortality of selected cancers by an area-based socioeconomic position index [24].

Identifying and addressing barriers to information access and resultant knowledge gaps is critical to prevent and rectify the existing excess burden of disease shouldered by vulnerable populations [10,11]. Although recent data showed that the Internet is the most frequently reported source of information among adults in Puerto Rico [17], factors associated with Internet use, and lack of use, have not been well documented in this population. Thus, our investigation aimed to assess use of the Internet, health information seeking, and cancer information seeking, and to explore differences in these behaviors between respondents who use the Internet and those who do not in a representative sample of adults in Puerto Rico. Our analyses also explored geographic variability in Internet use and health information seeking.

## Methods

The US National Cancer Institute developed the Health Information National Trends Survey (HINTS) as a surveillance mechanism for tracking population trends in cancer-relevant behavior, knowledge, and attitudes in the rapidly evolving health communication and informatics environment to inform effective health communication strategies across populations. In 2009, the University of Puerto Rico’s Comprehensive Cancer Center and the Department of Health in Puerto Rico–Puerto Rico Behavioral Risk Factor Surveillance System (BRFSS), in a collaborative effort supported by the National Cancer Institute, implemented a fully translated Spanish version of HINTS using existing BRFSS infrastructure in Puerto Rico [17]. The HINTS 2007 US mainland Spanish-language instrument, which contained response options and directions in English, was carefully reviewed, fully translated, and edited by the HINTS Puerto Rico team to ensure appropriateness for the island population. Greater details about the translation process have been reported elsewhere [17].

Data for this study were collected from April 27 through June 28, 2009 through random digit dial, computer-assisted telephone interview (N = 639). We used a stratified sampling frame to represent the eight geographic regions of the Puerto Rico Department of Health. Interviews were conducted primarily in Spanish by experienced bilingual Puerto Rican interviewers. The unweighted response rate for the screener and extended interview was 76.3% (837/1097).

### Measures

#### Sociodemographic Characteristics

Sociodemographic characteristics included the following: age (18–34, 35–49, 50–64, or 65+ years); gender; education (less than high school, high school graduate, some college or technical school, or college graduate and beyond); employment status (employed or not employed); annual household income (<US $15,000, $15,000–24,999, $25,000–34,999, or $35,000+); and marital status (married or living as married, or not married).

#### Access to Health Care and Health Status

To assess access to health care, respondents were asked if they had a regular source of health care and whether they had any kind of health insurance coverage. Respondents were also asked to rate their own health status on a scale ranging from poor to excellent.

#### Use of the Internet

Use of the Internet was assessed with the question “Do you ever go online to access the Internet or World Wide Web, or to send and receive email?” Responses were coded as yes or no.

#### Information Seeking

Respondents were asked the following questions about general health and cancer-specific information seeking: “Have you ever looked for information about health or medical topics from any source?” and “Have you ever looked for cancer information from any source?”

#### Geographic Location

**Figure 1:**
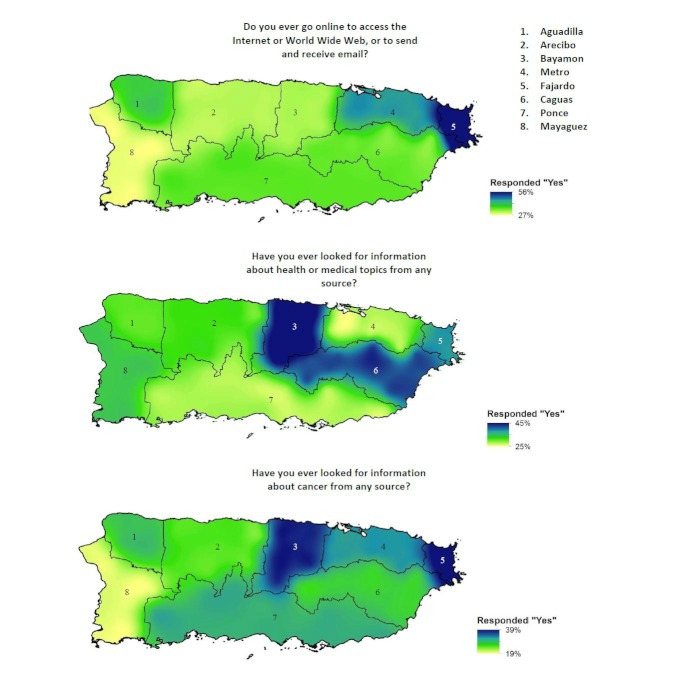
Geographic distribution of Internet use and health and cancer information seeking.

Geographic information was obtained through the sampling process and each respondent was placed into one of eight geographic regions in Puerto Rico: (1) Aguadilla, (2) Arecibo, (3) Bayamon, (4) Metro, (5) Fajardo, (6) Caguas, (7) Ponce, and (8) Mayaguez (see Figure 1).

### Data Analysis

To account for the complex sampling design and to calculate accurate standard errors, we used SUDAAN version 9.0.1 [28] for our analyses. All data were weighted according to key sociodemographic estimates from the US Current Population Survey to be representative of the population in Puerto Rico [29]. Frequencies were calculated for all measures. Cross-tabulation tables with chi-square tests of significance were conducted to identify significant bivariate associations of Internet use with information-seeking behaviors, sociodemographic characteristics, and health care access. Two multivariable logistic regression analyses were conducted including variables that were significantly associated with Internet use in the bivariate analyses to test for significant independent associations of health and cancer information seeking with Internet use while controlling for sociodemographic variables. Income was not included in the multivariable models because only 61.0% (390 of the total sample of 639) reported their income; inclusion of the income variable would have substantially reduced the sample size for the models.

We generated geographic information system maps to examine the geographic distribution of use of the Internet, cancer information seeking, and health information seeking on the island using a spline method of interpolation [30]. This interpolation method estimates values using a mathematical function that minimizes overall surface curvature, resulting in a smooth surface that passes exactly through the input points. We selected the spline method of interpolation to ensure stability and precision of calculations with the large sample size.

## Results

### Sociodemographic Characteristics

Table 1 summarizes weighted percentages and sample frequencies for sociodemographic characteristics and health care access for the total sample and by self-reported use of the Internet. Estimates summarized herein are weighed to be representative of the adult population in Puerto Rico; thus, direct calculation of percentages from the sample frequencies given does not equal the values given. Of 639 respondents, 450 (weighted percentage 53.7%) were female and 443 (weighted percentage 40.2%) were aged 50 years or over. Of the 600 respondents who reported their education, 430 (weighted percentage 76.0%) had at least a high school education. Only 390 of the total sample of 639 respondents reported their income level; of those 390 respondents, 117 (weighted percentage 35.9%) reported annual incomes of $25,000 or greater. Of 598 respondents reporting on their employment, 165 (weighted percentage 40.8%) were employed. Of 603 respondents, 310 (weighted percentage 48.5%) were married.

### Access to Health Care and Health Status

Of 636 respondents, 604 ( weighted percentage 92.1%) reported that they had health care coverage (either private or government-based coverage), and 507 of 637 respondents (weighted percentage 74.1%) reported having a regular source of health care. Of 604 respondents, 166 (weighted percentage 34.8%) rated their health as excellent or very good.

### Use of the Internet

Only 142 of 639 respondents (weighted percentage 32.7%) indicated that they ever went online to access the Internet. We observed several significant differences in the demographic profile of Internet users versus nonusers (Table 1). Generally, Internet users tended to be younger, to be more highly educated, to be employed, and to have higher annual incomes than non-Internet users. While fewer Internet users than nonusers reported having a regular health care provider, differences in health insurance status or perceived health were not statistically significant.

### Information Seeking

Table 1 also summarizes weighted frequencies for health and cancer information seeking for the total sample and by Internet use. Of 639 respondents, 219 (weighted percentage 32.9%) reported that they had ever looked for information about health or medical topics and 101 (weighted percentage 28.1%) reported that they had ever looked for information about cancer. Internet users engaged in health information seeking and cancer information seeking with over twice the frequency of non-Internet users. Among 142 Internet users, 101 (weighted percentage 59.6%) reported that they had ever looked for information on health or medical topics compared with 118 of 497 nonusers (weighted percentage 20.0%). The pattern was similar for cancer information seeking, wherein 76 of 142 Internet users (weighted percentage 47.2%) reported that they had ever looked for cancer information compared with 105 of 497 non-Internet users (weighted percentage 18.8%).

Table 2 summarizes results of two multivariable logistic regression models to test for significant associations of health and cancer information seeking with Internet access controlling for other variables that were significantly associated with Internet use in bivariate analyses (excluding income). Model 1 examined the association of having ever sought health or medical information with use of the Internet, controlling for age, education, employment, and regular source of health care. Model 2 examined the association of having ever sought cancer information with use of the Internet, controlling for age, education, employment, and regular source of health care. In both models, the same factors emerged as significant: information seeking, age, and education were independently and significantly associated with Internet use. The odds of using the Internet were over 5 times as likely (odds ratio 5.11, *P *< .001) among respondents who sought health information and 2.5 times as likely (odds ratio 2.5, *P *< .05) among respondents who sought cancer information. Age and education level were also independently and significantly associated with Internet use in both models, with Internet users being younger and more educated. The odds of using the Internet significantly decreased with each increase in specified age group and significantly increased with each increase in specified education level. These trends were confirmed in an analysis of the independent variable effects for both age and education, such that each of the age and education categories differed significantly from their respective referent categories in a linear fashion.

**Table 1:** Weighted estimates and sample frequencies for sociodemographic characteristics, health care access, and health information-seeking patterns of respondents who reported using the Internet and those who did not.

Characteristic		Total (n = 639)	“Do you ever go online to access the Internet or World Wide Web…?”		*P *value^a^
			Yes (32.7%) (n = 142)	No (67.3%) (n = 497)	
		%	%	%	
**Sex**		n = 639	n = 142	n = 497	
	Female	53.7	50.3	55.3	.44
	Male	46.3	49.7	44.7	
**Age range (years)**		n = 639	n = 142	n = 497	
	18–34	31.9	56.7	19.9	<.0001
	35–49	27.9	31.6	26.2	
	50–64	21.4	8.9	27.6	
	65+	18.7	2.9	26.4	
**Educational level**		n = 600	n = 136	n = 464	
	Less than high school	24.0	1.1	35.4	<.0001
	High school graduate	27.8	20.0	31.6	
	Some college	27.9	42.2	20.9	
	College graduate	20.3	36.7	12.2	
**Employment status**		n = 598	n = 135	n = 463	
	Employed	40.8	52.1	35.2	.01
	Not employed	59.2	47.9	64.8	
**Income ($US)**		n = 390	n = 94	n = 296	
	<15,000	36.9	18.5	46.3	<.0001
	15,000–24,999	27.2	23.2	29.3	
	25,000–34,000	14.4	16.3	13.4	
	35,000+	21.5	42.0	11.1	
**Marital status**		n = 603	n = 136	n = 467	
	Married/living as married	48.5	39.5	52.9	.07
	Not married	51.5	60.5	47.1	
**Regular health care provider**		n = 637	n = 142	n = 495	
	Yes	74.1	66.0	78.0	.047
	No	25.9	34.0	22.0	
**Health insurance coverage**		n = 636	n = 142	n = 494	
	Yes	92.1	87.7	94.2	.104
	No	8.9	12.3	5.8	
**Health status**		n = 604	n = 136	n = 468	
	Excellent or very good	34.8	46.6	29.0	.054
	Good	31.0	27.0	33.0	
	Fair or poor	34.2	26.5	38.0	
**Looked for health information**		n = 639	n = 142	n = 497	
	Yes	32.9	59.6	20.0	<.001
	No	67.1	40.4	80.1	
**Looked for cancer information**		n = 639	n = 142	n = 497	
	Yes	28.1	47.2	18.8	.0001
	No	71.9	52.8	81.2	

^a^ *P *values associated with chi-square tests of independence for sociodemographic, health care access, and information-seeking variables by Internet use.

**Figure 2:**
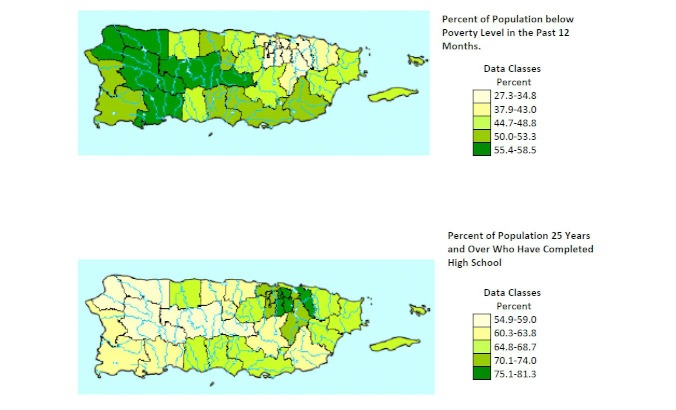
Geographic distribution of poverty and education in Puerto Rico. Data set: 2005-2009 Puerto Rico Community Survey 5-year estimates (public-use microdata).

**Table 2:** Multivariable logistic models of independent associations with Internet use.

Model			Odds ratio	95% CI^a^
**1**	**(n = 595), overall model ** *F* **9 ** **= 12.3, ** *P * **< .0001**			
	Looked for health information			
		No	1.00	1.00
		Yes	5.11	2.18–11.98
	Age range (years)			
		18–34	1.00	1.00
		35–49	0.31	0.13–0.70
		50–64	0.07	0.02–0.18
		65+	0.05	0.01–0.16
	Educational level			
		High school or less	1.00	1.00
		Some college	4.55	1.18–17.52
		College graduate or more	7.79	2.66–22.83
	Employment status			
		Employed	1.00	1.00
		Not employed	0.93	0.44–1.96
	Regular health care provider			
		No	1.00	1.00
		Yes	0.76	0.30–1.91
**2**	**(n = 595), overall model ** *F* **9 ** **= 15.4, ** *P * **< .001**			
	Looked for cancer information			
		No	1.00	1.00
		Yes	2.51	1.02–6.16
	Age range (years)			
		18–34	1.00	1.00
		35–49	0.30	0.13–0.70
		50–64	0.80	0.03–0.22
		65+	0.06	0.02–0.19
	Educational level			
		High school or less	1.00	1.00
		Some college	4.55	1.22–16.92
		College graduate or more	9.72	3.35–28.15
	Employment status			
		Employed	1.00	1.00
		Not employed	0.94	0.46–1.91
	Regular health care provider			
		No	1.00	1.00
		Yes	0.74	0.30–1.80

^a ^95% Confidence intervals (CIs) not containing 1 were considered statistically significant.

### Geographic Location

Figure 1 shows the geographic distributions of Internet use, and health and cancer information seeking. Geographic variability in Internet use and information seeking was observed, with relatively more frequent Internet use in the Fajardo and Metro regions than in other regions. The frequency of Internet use was lowest in the Mayaguez region. Health information seeking occurred most frequently in the Bayamon, Caguas, and Fajardo regions. The frequency of health information seeking was lowest in the Ponce and Metro regions. Cancer information seeking was most frequent in the Bayamon, Fajardo, and Metro regions, and least frequent in Mayaguez.

To place the observed geographic trends in Internet penetration into a context informed by related sociodemographic variables, we explored known geographic trends in key indicators of socioeconomic status: poverty and education. Figure 2 summarizes data from the American Community Survey (2005–2009) for poverty and education rates in Puerto Rico [29]. The highest levels of poverty and lowest levels of education were observed in Aguadilla, Arecibo, Ponce, and Mayaguez.

## Discussion

Only one-third of the population in Puerto Rico reported going online to access the Internet or World Wide Web in 2009. This finding is consistent with 2011 Internet penetration rates for Puerto Rico (37.3%) obtained through market research [31] and prior HINTS analysis documenting low use of the Internet among US Spanish-speaking Hispanics [1]. This percentage is considerably lower than the comparable estimate of 68.4% from HINTS 2008 data for the general population of the US mainland and 49% for Hispanics living in the US mainland [17,24] and well below the Healthy People 2020 goal of 75% [5]. Consistent with previous research, Internet users in Puerto Rico tended to be younger and more highly educated, to be employed, and to have higher annual incomes than non-Internet users [14,31].

Prior research analyzing HINTS data from Puerto Rico revealed that the population seems to be split in its degree of trust in the Internet as a source of health information [17]. About a quarter of the population in Puerto Rico indicated “a lot” of trust in the Internet as a source of health information, while a similar proportion indicated that they did not trust the Internet “at all” as a source of health information and the remaining half indicated “somewhat” or “a little” trust in the Internet. This distribution of responses to trust in the Internet is distinct from the distribution of responses to trust in other sources of mediated health information such as television and radio. While the percentage of the population indicating a lot of trust in the Internet is similar to that for television and radio (and several other sources), population trust in the Internet seems much more divided, with a much greater proportion of the population indicating not trusting the Internet at all than the percentage who indicated not trusting other sources of information. This finding speaks to the need to employ a range of outreach and educational mechanisms that emphasize use of traditional media and interpersonal sources of health information.

Health and cancer information seeking varied significantly between those islanders who accessed the Internet and those who did not. Persons who had sought health information and those who had sought cancer information had 5 times the odds and 2.5 times the odds, respectively, of using the Internet. These findings point to a potential digital divide and are consistent with previous research indicating that Hispanic populations are less likely to be health information seekers [1,4,17]. Although prior research has demonstrated that the most frequently identified source of health information in Puerto Rico is the Internet [17], overall Internet penetration on the island is fairly low, and a significant segment of the population does not trust the Internet as a source of health information [17], thereby limiting access to the growing body of online health and cancer information.

Patterns of information-seeking behavior by sociodemographic characteristics observed in this study are also consistent with those of prior research showing associations of cancer and health information seeking with educational attainment and age [4,17,32]. Overall, approximately one-third of the population in Puerto Rico had ever looked for information about health or cancer topics from any source; these findings are similar to those observed in the HINTS 2008 continental US Hispanic population (health, 50.9%; cancer, 26.4%) [17,33]. Moreover, cancer and health information-seeking behavior varied significantly between those who accessed the Internet and those who did not.

Geographic variability in Internet use and health and cancer information seeking was considerable on the island. Internet use was more frequent in the San Jaun metropolitan area (Fajardo and Metro regions) than on the rest of the island, where frequency of Internet use was substantially lower. Health information seeking occurred most frequently in the regions surrounding the San Juan metro area (Bayamon, Caguas, and Fajardo); curiously, health information seeking was somewhat lower in the Metro region proper. Cancer information seeking was most frequent in San Juan metro area (Bayamon, Fajardo, and Metro regions) relative to the more rural areas of the island. When comparing our results with demographic data from the Current Population Survey, we observed a pattern of a higher prevalence of Internet use in regions with a greater proportion of persons with higher educational attainment and a lower proportion of persons living below the poverty level. This pattern is consistent with the greater likelihood of Internet use observed in our models among persons with higher education.

The geographic patterns of Internet use and information seeking are consistent with prior research in Puerto Rico demonstrating socioeconomic disparities in incidence and mortality of selected cancers by an area-based socioeconomic position index [24]. Specifically, socioeconomic deprivation is greater in the centrally located municipalities of the island than in the municipalities in the San Juan metropolitan area [24]. It has been proposed that populations residing in the central mountainous regions of Puerto Rico face greater obstacles to health care, including economic, environmental, cultural, and social barriers [24]. Such barriers are likely shared in common with barriers to the Internet and health information.

Recognition of the geographic pattern of Internet access provides an excellent resource for cancer information communication planning on the island and can guide efforts to expand existing infrastructure to rural areas or promote adoption of mobile technologies to support health information seeking. Furthermore, these data can inform efforts to tailor information sources, including print, traditional, and new media, to varying geographic regions. The data also support the continued use of traditional and trusted sources of health information such as interpersonal communication between patient and doctor, and utilization of trusted and trained community health workers. These interpersonal communication strategies must be leveraged to ensure that those who are offline are connected to health information. They can also be used to point consumers to reliable and credible sources of online health information.

The following limitations are worth noting. The survey design for HINTS Puerto Rico is cross-sectional; therefore, definitive conclusions about causal associations are not appropriate. In this respect, it is important to note that the direction and nature of the association between Internet access and health information seeking cannot be determined from these data. Limitations are also inherent in the use of random digit dial telephone methods and self-report measures. Additionally, this study was a one-time snapshot of Internet usage during a time of rapid change in cell phone usage and advances in mobile technology. Over a third of the sample did not report their income; therefore, we excluded the income variable from the multivariable model, preventing us from examining the independent association of income with Internet use and information seeking. With current trends in penetration of cell phones with Internet access, the level of Internet usage has increased in Puerto Rico since the time of the study. Survey data collected in 2011 indicate that 44% of respondents who have a cell phone use it to surf the Web [34]. This number is double that of 2009, pointing to a rapid increase in use of mobile devices for Internet access [34]. Despite these limitations, the data were derived from a stratified representative sample from the eight geographic regions of the Puerto Rico Department of Health, providing an adequate sample size to enable “state”-level analyses. Furthermore, the response rates for this survey were quite high, lending further confidence to the generalizability of the findings.

Understanding health information-seeking behavior in relationship to use of the Internet is timely and important, given the rapid increase in the amount of information available online and the increasing influence of online health information seeking on health behaviors, health processes, and health outcomes [1,4,7,32,35]. Much of the content available online is published in English. Increasing Spanish-language content available online would remove a key barrier to access and likely increase use among Spanish-speaking populations. Allocation of resources to enable access to online information has considerable potential to reach a broad audience of consumers. Efforts to increase access to the Internet through expanding the urban infrastructure to rural regions and through use of mobile devices and applications are encouraged. In Puerto Rico, observation of distinct regions with low Internet penetration and information seeking (eg, Mayaguez) could help to inform both infrastructure (eg, broadband penetration in rural areas) and educational efforts to ensure that health information is made more available via traditional modalities. In addition to long-term planning around communication and health care infrastructure, health education and outreach efforts should consider using available and trusted [17] methods of dissemination such as radio and television, as well as community-based health care providers and organizations. Our analyses provide important insights into Internet use and health information-seeking behaviors and experiences of the population in Puerto Rico and contribute to the evidence base for health and cancer communication planning for the island.

## Abbreviations

BRFSS: Behavioral Risk Factor Surveillance System
HINTS: Health Information National Trends Survey

## References

[1] Viswanath K (2005). Science and society: the communications revolution and cancer control. Nat Rev Cancer.

[2] Beckjord EB, Finney Rutten LJ, Squiers L, Arora NK, Volckmann L, Moser RP, Hesse BW (2007). Use of the internet to communicate with health care providers in the United States: estimates from the 2003 and 2005 Health Information National Trends Surveys (HINTS). J Med Internet Res.

[3] Atkinson NL, Saperstein SL, Pleis J (2009). Using the internet for health-related activities: findings from a national probability sample. J Med Internet Res.

[4] Rutten LJ, Squiers L, Hesse B (2006). Cancer-related information seeking: hints from the 2003 Health Information National Trends Survey (HINTS). J Health Commun.

[5] Healthy People 2020 US Department of Health & Human Services.

[6] Shim M, Kelly B, Hornik R (2006). Cancer information scanning and seeking behavior is associated with knowledge, lifestyle choices, and screening. J Health Commun.

[7] Lustria ML, Smith SA, Hinnant CC (2011). Exploring digital divides: an examination of eHealth technology use in health information seeking, communication and personal health information management in the USA. Health Informatics J.

[8] Baker L, Wagner TH, Singer S, Bundorf MK (2003). Use of the Internet and e-mail for health care information: results from a national survey. JAMA.

[9] Czaja R, Manfredi C, Price J (2003). The determinants and consequences of information seeking among cancer patients. J Health Commun.

[10] Viswanath K (2006). Public communications and its role in reducing and eliminating health disparities. In: Thomson GE, Mitchell F, Williams MB, editors: Examining the Health Disparities Research Plan of the National Institutes of Health: Unfinished Business.

[11] Viswanath K, Breen N, Meissner H, Moser RP, Hesse B, Steele WR, Rakowski W (2006). Cancer knowledge and disparities in the information age. J Health Commun.

[12] Clayman ML, Manganello JA, Viswanath K, Hesse BW, Arora NK (2010). Providing health messages to Hispanics/Latinos: understanding the importance of language, trust in health information sources, and media use. J Health Commun.

[13] Fox S, Livingston G (2011). Latinos online.

[14] Hesse BW, Nelson DE, Kreps GL, Croyle RT, Arora NK, Rimer BK, Viswanath K (2005). Trust and sources of health information: the impact of the Internet and its implications for health care providers: findings from the first Health Information National Trends Survey. Arch Intern Med.

[15] Livingston G, Parker K, Fox S (2006). Latinos Online-2008: narrowing the gap.

[16] Vanderpool RC, Kornfeld J, Rutten LF, Squiers L (2009). Cancer information-seeking experiences: the implications of Hispanic ethnicity and Spanish language. J Cancer Educ.

[17] Tortolero-Luna G, Finney Rutten LJ, Hesse BW, Davis T, Kornfeld J, Sanchez M, Moser RP, Ortiz AP, Serrano-Rodriguez RA, Davis K (2010). Health and cancer information seeking practices and preferences in Puerto Rico: creating an evidence base for cancer communication efforts. J Health Commun.

[18] Ortiz AP, Soto-Salgado M, Calo W, Nogueras G, Tortolero-Luna G, Hebl S, Figueroa-Vallés N, Suárez E (2010). Disparities in breast cancer in Puerto Rico and among Hispanics, non-Hispanic whites, and non-Hispanics blacks in the United States, 1992-2004. Breast J.

[19] Ortiz AP, Soto-Salgado M, Calo WA, Tortolero-Luna G, Pérez CM, Romero CJ, Pérez J, Figueroa-Vallés N, Suárez E (2010). Incidence and mortality rates of selected infection-related cancers in Puerto Rico and in the United States. Infect Agent Cancer.

[20] Ortiz AP, Pérez J, Otero-Domínguez Y, García-Rodríguez O, Garced-Tirado S, Escalera-Maldonado F, Gaud-Quintana S, Santiago-Rodríguez E, Svensson K, Vergara-Arroyo JL, Ortiz K, Torres M, Tortolero-Luna G, Figueroa-Vallés N (2010). Endometrial cancer in Puerto Rico: incidence, mortality and survival (1992-2003). BMC Cancer.

[21] Romero Marrero C, Ortiz AP, Pérez CM, Pérez J, Torres EA (2009). Survival of hepatocellular carcinoma in Puerto Rico. P R Health Sci J.

[22] Soto-Salgado M, Suárez E, Calo W, Cruz-Correa M, Figueroa-Vallés NR, Ortiz AP (2009). Incidence and mortality rates for colorectal cancer in Puerto Rico and among Hispanics, non-Hispanic whites, and non-Hispanic blacks in the United States, 1998-2002. Cancer.

[23] Suárez E, Calo WA, Hernández EY, Diaz EC, Figueroa NR, Ortiz AP (2009). Age-standardized incidence and mortality rates of oral and pharyngeal cancer in Puerto Rico and among Non-Hispanics Whites, Non-Hispanic Blacks, and Hispanics in the USA. BMC Cancer.

[24] Torres-Cintrón M, Ortiz AP, Ortiz-Ortiz KJ, Figueroa-Vallés NR, Pérez-Irizarry J, Díaz-Medina G, De la Torre-Feliciano T, Suárez-Pérez E (2012). Using a socioeconomic position index to assess disparities in cancer incidence and mortality, Puerto Rico, 1995-2004. Prev Chronic Dis.

[25] Hugo MH, Velasco G (2011). A demographic portrait of Puerto Ricans, 2009.

[26] Rivera-Hernandez N, Andino-Ortiz VL (2011). National Council of La Raza.

[27] Krieger N, Chen JT, Waterman PD, Soobader MJ, Subramanian SV, Carson R (2002). Geocoding and monitoring of US socioeconomic inequalities in mortality and cancer incidence: does the choice of area-based measure and geographic level matter?: the Public Health Disparities Geocoding Project. Am J Epidemiol.

[28] Research Triangle Institute RTI International.

[29] (2010). US Census Bureau.

[30] Kaw AK, Kalu EE, Besterfield G (2008). Numerical Methods with Applications: 2nd edition.

[31] Internet World Stats (2011). Miniwatts Marketing Group.

[32] Fox S (2006). Online Health Search 2006.

[33] Health Information Trends Survey National Cancer Institute.

[34] SMEI home page Sales & Marketing & Executives International, Inc.

[35] Lambert SD, Loiselle CG (2007). Health information seeking behavior. Qual Health Res.

